# Mealtime, Temporal, and Daily Variability of the Human Urinary and Plasma Metabolomes in a Tightly Controlled Environment

**DOI:** 10.1371/journal.pone.0086223

**Published:** 2014-01-24

**Authors:** Kyoungmi Kim, Christine Mall, Sandra L. Taylor, Stacie Hitchcock, Chen Zhang, Hiromi I. Wettersten, A. Daniel Jones, Arlene Chapman, Robert H. Weiss

**Affiliations:** 1 Division of Nephrology, Department of Internal Medicine, University of California, Davis, Davis, California, United States of America; 2 Division of Biostatistics, Department of Public Health Sciences, University of California, Davis, Davis, California, United States of America; 3 Cancer Center, University of California, Davis, Davis, California, United States of America; 4 Division of Nephrology, Department of Internal Medicine, Emory University, Atlanta, Georgia, United States of America; 5 Department of Chemistry, Michigan State University, East Lansing, Michigan, United States of America; 6 Department of Biochemistry and Molecular Biology, Michigan State University, East Lansing, Michigan, United States of America; 7 Medical Service, Sacramento VA Medical Center, Sacramento, California, United States of America; University College Dublin, Ireland

## Abstract

While metabolomics has tremendous potential for diagnostic biomarker and therapeutic target discovery, its utility may be diminished by the variability that occurs due to environmental exposures including diet and the influences of the human circadian rhythm. For successful translation of metabolomics findings into the clinical setting, it is necessary to exhaustively define the sources of metabolome variation. To address these issues and to measure the variability of urinary and plasma metabolomes throughout the day, we have undertaken a comprehensive inpatient study in which we have performed non-targeted metabolomics analysis of blood and urine in 26 volunteers (13 healthy subjects with no known disease and 13 healthy subjects with autosomal dominant polycystic kidney disease not taking medication). These individuals were evaluated in a clinical research facility on two separate occasions, over three days, while on a standardized, weight-based diet. Subjects provided pre- and post-prandial blood and urine samples at the same time of day, and all samples were analyzed by “fast lane” LC-MS-based global metabolomics. The largest source of variability in blood and urine metabolomes was attributable to technical issues such as sample preparation and analysis, and less variability was due to biological variables, meals, and time of day. Higher metabolome variability was observed after the morning as compared to the evening meal, yet day-to-day variability was minimal and urine metabolome variability was greater than that of blood. Thus we suggest that blood and urine are suitable biofluids for metabolomics studies, though nontargeted mass spectrometry alone may not offer sufficient precision to reveal subtle changes in the metabolome. Additional targeted analyses may be needed to support the data from nontargeted mass spectrometric analyses. In light of these findings, future metabolomics studies should consider these sources of variability to allow for appropriate metabolomics testing and reliable clinical translation of metabolomics data.

## Introduction

Of the growing number of omics techniques in current use in basic and clinical science, metabolomics is most closely related to organismal phenotype [1], [2]. With this technique, all of the metabolites produced endogenously (and sometimes exogenously) in a living organism are analyzed in order to ascertain the internal biochemical and metabolic processes taking place, such that insights regarding normal physiology and pathophysiology can be gleaned. To date, there have been many inroads using metabolomics in medicine, such that the field is now poised to discover clinically useful biomarkers and therapeutic targets in nephrology, cancer, and other medical fields (reviewed in [2]–[4]).

However, despite these advances, a healthy dose of caution is required with respect to clinical translation in light of some widely publicized premature attempts (discussed in [5]). Given the relatively recent and rapid proliferation of clinical human studies on metabolomics, it is essential that due diligence be considered with regard to the terms of limitations of data interpretation, such that future studies, and their conclusions, as well as clinical applications are robust, reproducible, reliable and informative. For a single (or a suite of) metabolite biomarker(s) to be useful as a diagnostic or prognostic screening tool, its variability as a function of time of day, dietary exposure, exercise, medication use, smoke exposure and other exogenous stimuli needs to be established and quantified such that interpretation of the data is reliable and accurate. For example, one would not like to perform a cancer biomarker study only to find that what is identified as a metabolomics signal is a function of time after a meal or due to a medication exposure, rather than as a result of the underlying biology of the cancer of interest [6].

While there have been several human and animal studies attempting to address these issues, there have been very few evaluations of metabolome variability in human subjects under tightly controlled conditions in clinical settings [7], [8], and even fewer studies have evaluated all relevant parameters concurrently in both blood and urine [7]. While there have been attempts to evaluate normal individual metabolomes [9], [10] demonstrating the presence of personal metabolomic phenotypes [11], there have been no published studies analyzing the sources of variability utilizing blood and urine in an inpatient environment in the setting of a chronic stable disease. In addition, most of the available studies on diet and metabolomics have focused on the effects of a specific dietary intervention [12]–[14] rather than time-of-day or day-to-day variability. Therefore the present study was undertaken utilizing healthy volunteer subjects (half with a chronic stable hereditary kidney disorder) who were admitted to an inpatient clinical research center and given standardized meals with regard to calorie and carbohydrate intake at a consistent time of day while blood and urine samples were obtained under uniform conditions at specific times. Following sample procurement, samples were handled in standardized fashion for both urine and blood samples, and metabolomics data were subjected to rigorous statistical evaluation. An extensive analysis of the data was performed to identify intrinsic sources of variability as well as the nature of the mealtime and temporal changes that occur within these two readily accessible biofluids. Incorporating these findings into future blood and urine based metabolomics studies will lead to considerably sounder study conclusions.

## Materials and Methods

### Ethics statement

The study was approved by the Institutional Review Boards both at Emory University and at the University of California, Davis. All participants gave written informed consent.

### Subject enrollment

To qualify for the study, participants had to be without any medical condition (with the exception of ADPKD in that cohort), not be taking any prescription medications or over the counter medications within two weeks of study; and for women, have regular menstrual cycles or be post-menopausal. In addition, all clinical laboratory analyses including measurement of hematopoietic and kidney function were required to be within normal limits. Thirteen healthy volunteers and 13 subjects diagnosed with ADPKD according to the Pei et al criteria [15] (Table S1) were recruited and admitted to the inpatient unit of the Clinical Research Network (CRN) of the Atlanta Clinical and Translational Science Institute at Emory University Hospital. Subjects did not take prescription or over the counter medications for a minimum of 2 weeks prior to study with the exception of one subject, who had taken St. John's Wort, vitamin E and black cohosh within one day of evaluation. All female subjects who were menstruating were evaluated in the follicular phase of the menstrual cycle to eliminate variability related to post-ovulatory changes. All subjects were interviewed by the research dietician of the CRN and weighed. Based on the subject food preferences and allergies a standardized test meal was developed by the Bionutrition unit of the Clinical Research Network (CRN) that provided similar calories as a function of body weight with breakfast, lunch, and dinner, each prepared in identical fashion for each day of testing. The 24-hour calorie intake prescribed was 2,500 kcal/kg/day and 1.1 g/kg/day protein intake. Subjects were admitted to the CRN for two days of testing (Days 1 and 2) and on a separate occasion for Day 3 of testing. During this time period, subjects were fed the pre-prepared standardized meals and fluids at set times of day. Subjects were asked to consume all foods on their tray within a specified period of time. Following completion of the meal, the research dietician retrieved the food trays and kept all unfinished foods for analyses. Following the initial two-day study, the subjects were admitted again for another half day of testing (Day 3). All subjects were fed the same standardized meals as those served on Day 1 and 2 at the same time of day, blood and urine samples were drawn at the same time (see Study Design in Table 1) and then handled similarly by the same person (S.H.), frozen at −80°C within 30 min. All sample collection tubes were from a single batch and plasma was separated from whole blood before freezing. Samples were shipped on dry ice to the analytical laboratories for metabolomics analysis.

**Table 1:** Study Design: Times of urine and blood collection and meals.

Day 1	Day 2	Day 3
+1 hour (Fasting Pre Breakfast)	+1 hour (Fasting Pre Breakfast)	+1 hour (Fasting Pre Breakfast)
08:30	08:30	08:30
09:00 Meal	09:00 Meal	09:00 Meal
+3 hour (Post Breakfast)	+3 hour (Post Breakfast)	+3 hour (Post Breakfast)
11:00	11:00	11:00
12:00 Meal		
+7 hour (Post Lunch)		
15:00		
+9 hour (Pre Dinner)		
17:00		
17:30 Meal		
+11 hour (Post Dinner)		
19:30		
+14 hour (Late Night)		
21:30		

### Non-targeted metabolomic analysis

For these studies, the chemical identification of only a subset of metabolites was performed, since compound identity is not required for evaluating pure metabolome variability. For logistical reasons, the urine and blood analyses were performed in two distinct state-of-the art laboratories; however, the experimental design was identical for the entire course of the metabolomics analyses.

#### Plasma sample preparation

Frozen plasma samples were thawed at room temperature, and a 10-µL aliquot was removed from each tube and transferred to a polypropylene microcentrifuge tube containing 90 µL of HPLC-grade methanol. These mixtures were centrifuged at 10,000×*g* and 4°C for 10 minutes, and 50 µL of each supernatant and 50 µL of Milli-Q water were transferred to an amber autosampler vial containing a low volume glass insert. The samples were organized based on a pre-determined order of analysis, including a series of technical replicates analyzed with each 54-vial tray of plasma extracts.

#### Plasma metabolite profiling

Plasma metabolites were profiled using liquid chromatography/time-of-flight mass spectrometry (LC/TOF-MS) using a LCT Premier mass spectrometer (Waters Corp., Milford, MA) that was coupled to three LC-20AD pumps, column oven, and a SIL-5000 autosampler (Shimadzu, Columbia, MD) equipped with a six-tray chilled autosampler stack that was held at 10°C. Aliquots (10 µL) of each extract were injected, and metabolites were separated using an Ascentis Express C18 column (2.1×50 mm; 2.7 µm particles; Sigma-Aldrich, St. Louis, MO) and linear gradient elution based on solvents A (0.15% aqueous formic acid), B (acetonitrile/methanol 1∶1 v/v), and C (acetone) as follows (A/B/C): 0–1.0 min (95/5/0), 1–3 min (program to 40/60/0). 3–9 min (program to 0/100/0), hold until 11 min. At 11 min, the solvent was programmed to 100% C at 12 min to wash the column and this composition was held until 13.5 min, after which the solvent was returned to the initial condition. Total flow rate was 0.40 mL/min, and the column was held at 50°C. Mass spectra were acquired using electrospray ionization (data were acquired in both positive and negative modes separately), over *m/z* 50–1500. Quasi-simultaneous acquisition of mass spectra was performed in four separate acquisition functions using W-mode ion optics (mass resolution of 10,000), transit lens voltage settings of 10, 30, 50, and 75 volts, and spectrum acquisition time of 0.15 seconds per function. Only data acquired under the first acquisition function were processed during this study, but the higher energy functions yielded fragment ions useful for metabolite annotation [16], and this process was aided through manual comparison of molecular and fragment masses with entries in the Human Metabolome Database [17].

LC/TOF-MS data were processed using Waters MarkerLynx software, which performs peak detection, integration, and retention time alignment. Processed results were exported as unscaled peak areas, organized by chromatographic retention time and *m/z* (mass) value, into a Microsoft Excel spreadsheet.

#### Urine sample preparation

Urine sample aliquots kept at −80°C were thawed on ice and 150 µL of ice-cold methanol was added to each sample [18]. The volumes of the urine samples were normalized to osmolality. Samples were vortexed vigorously then placed at −20°C for 1 hour in a pre-chilled tube rack. Samples were then vortexed briefly and microcentrifuged for 10 minutes at max speed. Supernatants were removed and transferred to a clean microcentrifuge tube while leaving pellet undisrupted. Supernatant was microcentrifuged again at max speed and once again transferred into a clean microcentrifuge tube. Samples were then subjected to SpinVac to dryness with heating to 60°C or less for approximately 3 hours. Samples were then resuspended in 50 µL of water: acetonitrile (98∶2) and centrifuged for 5 minutes in microcentrifuge at max speed and transferred to plastic autosampler tube and capped. Samples were stored at 4°C if used within 1–2 days otherwise kept at −20°C.

#### Urine metabolite profiling

HPLC-MS analysis was performed on an Agilent 1200 Series Autosampler coupled to an Agilent 6530 Accurate Mass Q-TOF with an Agilent Jet Stream electrospray ionization source. Analytes were separated on a Phenomenex Kinetex 2.6 µm XB-C18 100A reverse phase column using 0.1% formic acid in water (A) and 0.1% formic acid in acetonitrile (B). The gradient was run at 0.35 ml/minute and consisted of an isocratic separation for 0.5 minutes of 2% B, and then B was increased at a linear rate to 70% at 13 minutes, 95% at 14 minutes, held at 95% until 15 minutes, then re-equilibrated for 3 minutes with 2%B at 0.5 ml/min. The mass spectrometer was set to measure ions over *m/z* 60–1000 in positive ionization mode at a rate of 6 spectra per second. Automatic MS/MS was enabled with a scan rate of 6 spectra per second and a range of *m/z* 25–1000. MS/MS active exclusion was enabled and collision energy was set to range from 20–70 eV. Agilent Mass Hunter Qualitative Analysis was used to export raw peak areas from the RP-HPLC-QToF using the “find by molecule” feature. The data were then exported as a CEF file to Agilent Mass Profiler Professional for peak alignment. Retention time window was set to +/−0.5 min and there was no minimum area cutoff. This aligned peak list was then exported for recursive analysis using the “export for recursion” function by Agilent Mass Profiler Professional. Recursive analysis was then performed, which is a more targeted approach to fill in the missing values, using the “find by formula” feature in Agilent Mass Hunter Qualitative analysis with known retention times. The peaks were then re-exported to Agilent Mass Profiler Professional and re-aligned. The filter by frequency function was used and set to 60% of all samples. Raw data were then exported to a CSV file and put into Microsoft Excel. A compound database was made using MS/MS matches from NIST, ReSpect, LipidBlast, Metlin AM, and manual search by accurate mass and MS/MS features found in samples. Retention times and formulas were recorded and put into the database. The “find by formula” feature in Agilent Mass Hunter Qualitative Analysis was used with retention time matching +/−0.3 minutes. Compound peaks were then exported as CEF files to Mass Profiler Professional and aligned. Raw data were then exported to Microsoft Excel.

### Statistical Analysis

Six plates were required to analyze all samples using the LC-MS equipment for both urine and plasma; placement of samples on each plate was randomly determined. To investigate batch effects potentially resulting from the need to use six plates, we randomly selected 3 control and 3 ADPKD subjects' fasting samples collected on Day 2 (urine and blood analyzed separately) to serve as “reference” quality control samples; each of these reference samples was analyzed on every plate analyzed on the LC-MS platforms. Batch effects and within-lab reproducibility were evaluated using the repeated measurements of the six reference samples included on all plates.

A total of 782 urinary metabolites and 873 blood metabolites were identified in at least one sample. Among them, only the 294 urinary metabolites and 121 blood metabolites were detected in all 296 samples (26 subjects×10 time points plus 36 reference samples) analyzed. Because this study focused on variability and imputing missing values could artificially affect the variability, we limited our analyses to the 294 urinary metabolites and 121 plasma metabolites that were detected in all samples to avoid the bias that could be induced by imputation. For normalization, the intensity values for each sample run were summed, and then the median value of the sums across all samples was determined. The intensity values of each sample were scaled such that the sum of the scaled intensities equaled the median value. Thus, the sum of the scaled intensities was the same for all samples. Normalized intensity values were then log_2_ transformed to meet assumptions of normality and homoscedasticity of statistical tests and reduce the influence of extreme values.

We conducted variance component analyses (VCA) to estimate the relative contributions of the temporal factors (meals, time of day, and day-to-day effects) to the total variation in metabolite intensities. To assess meal effects, we restricted the analysis to samples collected before and after breakfast (hours +1 and +3; Table 1) and before and after dinner (hours +9 and +11). Meal effects were separately analyzed for each day. For day-to-day effects, we used samples repeatedly collected over 3 days at each time point before and after breakfast (i.e., at hour +1 and hour +3 separately). Finally, time of day effects were evaluated using all samples collected on Day 1 with time of day modeled as a categorical variable. A VCA was conducted for each metabolite. Specifically, the intensity level of the each metabolite, *Y_i_*, were modeled as follows: *Y_i_ = μ_i_+TempFactor_i_+Subject_i_+ε*_i_, where *TempFactor∼N(0, σ^2^_F_)* is the effect of each temporal factor (meal, time-of-day, and day-to-day) variation among measurement units; *Subject∼N(0, σ^2^_S_)* is the effect of subject variation among measurement units; and *ε_i_∼N(0, σ^2^_R_)* is the residual error, i.e., variation caused by factors other than the variables included in the model. All factors were modeled as random effects. Given the small sample size of the study population relative to the number of variables, we did not evaluate age, gender, race or ADPKD effects. Instead, all subject characteristics (age, gender, race, PKD status, etc.) were encompassed by the “subject” component to quantify the subject-to-subject variability. For each metabolite, variance components were estimated by the relative proportion of the total variation of the metabolite contributed by each temporal factor (meal, time-of-day, and day-to-day), subject and residual error. The total variance was assumed to be the sum of three components: *VAR_Tot_ = VAR_TempFactor_+VAR_Subject_+VAR_Residual_*. The relative proportion of each source of variation was calculated as a ratio of the variance estimate to the sum of all three variance estimates. For example, 
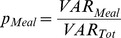
 calculates the proportion of the temporal factor variation attributed to meal and 
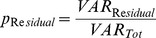
 calculates the proportion of variation due to unaccounted variation (residual error).

We also performed differential analyses to identify individual metabolites whose intensities were altered significantly in relation to the temporal factors. We used a mixed effects analysis of variance (ANOVA) for the differential analyses. The intensity of each metabolite was modeled as a function of the temporal factor of interest (meal, time-of-day, and day-to-day). A random subject effect was included to account for the correlation of repeated measurements from the same subject. False discovery rates (FDRs) were calculated to adjust for multiple testing and a FDR rate <0.05 was considered significant. Variance component and differential analyses were conducted with Proc Mixed in SAS version 9.2 (SAS Institute, Cary, NC).

## Results

### Subject population and protocol conduct

Subjects were studied on the inpatient unit of the Clinical Research Network of the Atlanta Clinical and Translational Institute between March and October, 2010. Characteristics of the subjects are provided in Table S1. Of note these were relatively young (33+/−10.9 years), non-obese (BMI 24.3+/−3.3 m^2^) subjects with 12 men and 14 women (58% Caucasian). Mean eGFR based on the MDRD formula was 99+/−17 mls/min/1.73 m^2^. Women were studied between days 5–13 of their menstrual cycle. All breakfasts were served between 8:57 am and 9:35 am, all lunches between 12:00 and 12:05, and all dinners between 17:29 and 17:55 (Table 1). One hundred percent of 123/130 meals were consumed with the remaining seven meals being completed by more than 91% of gms provided. Average measured dietary intake was 2620+/−560 kcal/kg/day and protein intake was 1.04+/−0.3 gm/kg/day.

### Reproducibility of metabolomics data: batch effects

The rationale for using both normal, healthy individuals as well as an identical size cohort of subjects with a chronic stable kidney disease (ADPKD) with normal kidney function was to be able to ultimately extend our findings to biomarker studies of this and other diseases. However, the subsequent data were also evaluated separately for these two cohorts and showed qualitatively similar results (File S1), confirming the utility of our analysis for both normal and diseased states. The metabolites analyzed were limited to those present in all samples, as our study was not designed to discover metabolite biomarkers for PKD.

All urine and plasma samples were separately processed from the participants by LC/TOF-MS in one equipment run (albeit there were separate plates for each run as necessitated by several day operation, see below) for each biofluid (Files S2a [plasma] and S2b [urine], uploaded into MetaboLights). However, because of the high number of samples (296) and the consequent necessity to process the samples in several batches (i.e. plates), it was considered possible that this introduced variability into the data. Thus, batch effects on the metabolome (defined as the systematic error introduced by time and plate-dependent variations prior to specific metabolome variability analyses) were evaluated by including six reference biofluid samples on each plate. For the assessment of batch effects, intensity values were log_2_ transformed but not normalized, since normalization could preclude discovery of systematic shifts between plates. Boxplots of the intensity values of each reference sample on each plate did not reveal any large, systematic pattern in the magnitude or distribution of intensities across batches (Figs. S1, S2). Scatter plots for each reference sample showed high correlations between intensities measured on different plates (data not shown). We calculated the coefficient of variation (SD/mean) across the six plates for each metabolite in each reference sample to provide a direct quantitative measure of technical between-batch variability. The median CV values of the 6 samples ranged from 0.012 to 0.097 and 0.070 to 0.110 in urine and blood, respectively (Fig. S3, S4). These low CV values demonstrate adequate technical reproducibility of sample analyses across all of the six plates used for the LC/TOF-MS analyses. Further, the results demonstrate that systematic batch effects were minimal and that, at a gross level, the LC/TOF-MS procedures that required running multiple plates for both analyses (plasma and urine) yielded reproducible results.

### Source and proportion of variation attributable to each effect

For a metabolite biomarker, or suite of metabolite markers, to be clinically useful, it is critical to define sources of variation of the metabolome that could result in changes in the metabolite concentration and thereby confound the clinical interpretation. For example, if the metabolite signal changes as a function of meal or collection times, specific sample procurement procedures will need to be implemented in order to eliminate variability due to influences other than the factor of interest (e.g. disease state), and to avoid bias which can occur during the process of specimen collection. In this study, we focused on assessing the influence on the metabolome of environmental and physiological factors related to meals and time of day of sample collection, because these are clinically relevant parameters with respect to sample selection and collection.

**Figure 1:**
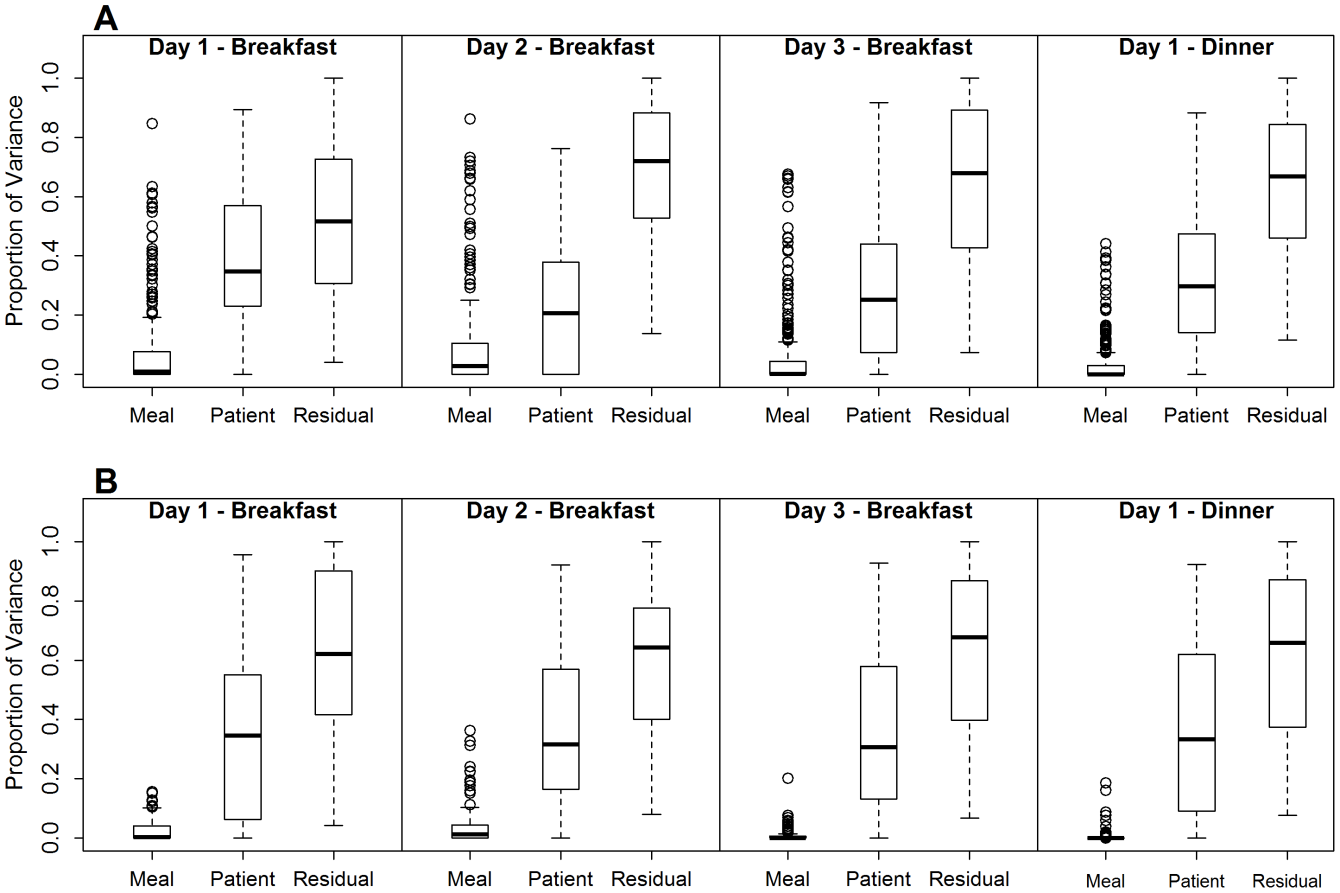
Variance components analysis in urine (A) and blood (B) metabolites for meal effects. Distribution of the relative proportion of total variance explained by each factor (meal, subject, residual) across all metabolites. Data of Hours +1 (pre-) and +3 (post- breakfast) and Hours +9 (pre-) and +11 (post-dinner) in each day were used in the analysis to estimate the proportion of variation attributable to meal effects for each of individual metabolites separately.

**Figure 2:**
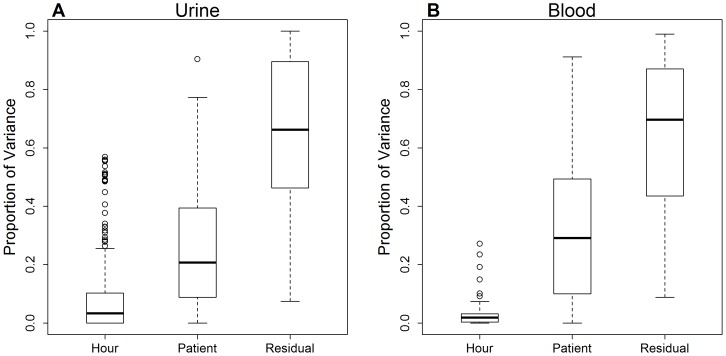
Variance components analysis in urine (A) and blood (B) metabolites for time of day effects. Distribution of the relative proportion of total variance explained by each factor (time of day in hour, subject, residual) across all metabolites. For time of day effects, data measured at hour +1, +3, +7, +9, +11, and +14 on Day 1 were used in the analysis to estimate the proportion of variation attributable to time of day effects for each of individual metabolites separately.

Using VCA, we quantified the relative amount of variation in the metabolomic data arising from the following clinically important factors: mealtime, time-of-day, day-to-day, subject-to-subject, and residual variability. It is important to keep in mind that we are looking at the *relative* amount of variability attributed to each source of variance proportional to the total variability in the data (total variability = 100%), since the *absolute* quantitation of variability varies by experiment and mass spectrometer used for each run. The largest percentage source of variability in this study was attributable to “residual variability,” which accounted for >50% of the observed variation in intensity for most of the metabolites (Figs. 1 and 2). This form of variability is a result of technical variability (i.e., the multiple step process of sample preparation and LC/TOF-MS analysis) and possibly experimental variability (i.e., sample collection, although this was very unlikely in this study due to the high consistency in the collection procedures) rather than intrinsic biological variability. Although less than technical and experimental variability, between subject variability, which represents intrinsic biological properties of the samples (i.e., genetic or metabolic differences among subjects), was also large across the subject population and may have been in part related to gender and ethnic heterogeneity and general health status among the human subjects. This data suggest that minimizing technical variability is equally important to increasing a sample size to yield significant findings for a clinically applicable human biomarker study.

To evaluate the effect of meals on metabolome variability, we obtained blood plasma and urine from the subjects under tightly controlled conditions in an inpatient clinical research facility using prepared meals with standardized components for equivalent carbohydrate and calorie intake normalized to BMI. Samples were obtained at fasting (+1 hr) and 1 hour post-breakfast (+3 hr) as well as 1 hour before (+9 hr) and after dinner (+11 hr; Table 1). Repeated samples taken from the same subject before and after the morning and evening meals showed a surprisingly low degree of variation attributable to meals as compared to other factors. In addition, variation attributable to meals was higher in urine than in blood (“meal” bars in Fig. 1). Interestingly, for the majority of the metabolites, the meal effects on the metabolome variability were more pronounced in the morning than in the evening meal, indicating that the impact of eating after the approximately 8 hours fast and sleep overnight on degree of changes in the metabolome was more evident than that of eating after a short fast of 4 hours during an awake and active period (compare “Day 1-, 2-, 3-Breakfast” with “Day 1-Dinner” in Fig. 1).

The between day comparison of each fasting to post-breakfast sample over 3 days demonstrated that the variability attributed to meals was relatively consistent in both urine and blood (Fig. 1), indicating reasonable inter-day reproducibility of the metabolite intensity, an important consideration for reproducibility in any future metabolomics-based biomarker test. To further assess inter-day variability as a function of meals, the coefficient of variation (CV) was calculated for each metabolite across three days for fasting samples and post-breakfast samples. The distributions of the CVs were very similar for fasting and post-breakfast samples (Fig. S5). Thus, both fasting and post-breakfast meal samples had similar inter-day reproducibility and inter-day variability was not prone to variability due to meals.

The estimated proportions of total variability attributed to each temporal factor across all metabolites are summarized in Table 2. The percentage of the variance accounted for by the morning meal varied among urinary metabolites, ranging from 0 to 84.6% with an average of 7.6% on Day 1, from 0 to 86.1% with an average of 8.7% on Day 2, and from 0 to 67.6% with an average of 6.2% on Day 3. The evening meal accounted for less variation than the breakfast meal and ranged from 0 to 44.2% with an average of 3.6% on Day 1. Consistent with previously discussed biofluid differences, the meal effects were nearly half or lower in blood as compared to urine, with a maximum percentage of 36.3% on Day 2.

**Table 2:** Proportion (mean ± SD) of variance attributable to each source of variation relative to the total variation in metabolite intensity across all metabolites.

		Within-Day Variability					Between-Day Variability
	Source of variance	Meal effects				Time of day effects	Day-to-Day effects
		Day 1	Day 1	Day 2	Day 3	Day 1	Days 1–3
		(+1 &+3)	(+9 &+11)	(+1 &+3)	(+1 &+3)	(hours +1 to +14)	(only +1 hour)
	Meal	0.076±0.14	0.036±0.08	0.087±0.15	0.062±0.13	0.084±0.13	0.023±0.039
Urine	Patient	0.396±0.22	0.306±0.21	0.228±0.21	0.28±0.24	0.249±0.19	0.306±0.17
	Residuals	0.528±0.24	0.658±0.22	0.685±0.23	0.658±0.25	0.667±0.22	0.670±0.18
	Meal	0.025±0.04	0.007±0.03	0.041±0.07	0.009±0.02	0.027±0.04	0.01±0.02
Blood	Patient	0.35±0.28	0.37±0.29	0.36±0.24	0.362±0.29	0.322±0.26	0.287±0.24
	Residuals	0.625±0.27	0.623±0.29	0.599±0.23	0.628±0.29	0.651±0.26	0.703±0.24

**Figure 3:**
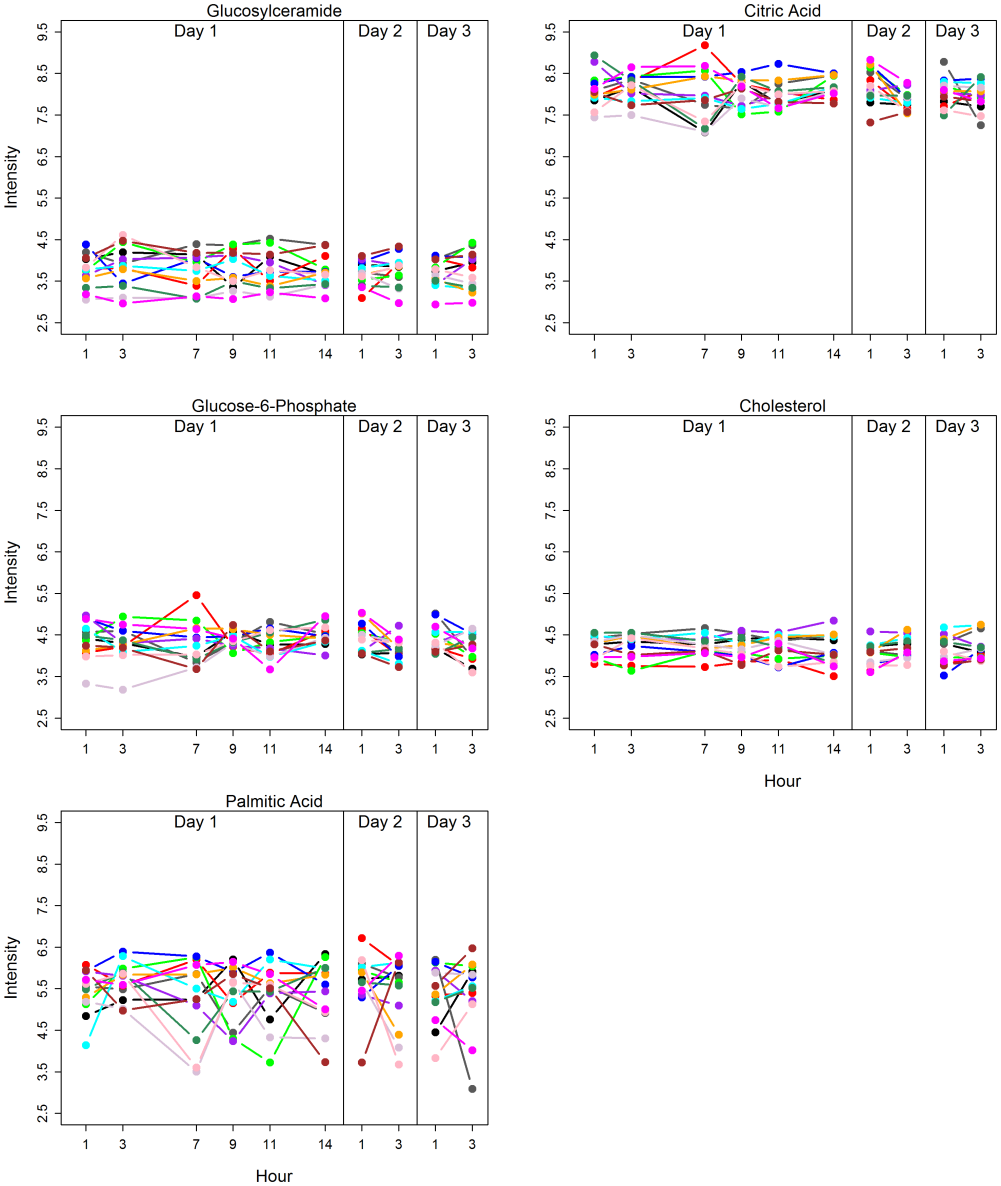
Temporal variability of selected metabolites. Variability in intensity values (log_2_ transformed) of selected metabolites in healthy volunteers is graphed as a function of indicated time (see Table S2). Each colored line represents one subject.

Metabolite intensities showed some variability as a result of time of day, which was partially confounded by meal effects (Fig. 2) and was also considerably lower in blood than in urine. The correlations in metabolite intensities were calculated between each pair of measurements at two time points on Day 1. In urine, the correlations between repeated measurements were larger for nearby times than far-apart times (Table 3), however, this did not occur with the blood metabolome. The correlation coefficients were constant regardless of the length of time interval between the measurements in blood (Table 4), demonstrating that the blood metabolome was less influenced by meal or time of day of sample collection than was the urine metabolome. We also observed that the intraday temporal patterns of metabolite intensity change over time varied by individual subject and by metabolite (Fig. S6), indicating the presence of complex heterogeneity of metabolite changes among subjects. Figure 3 illustrates the temporal patterns of variability of specific blood metabolites selected as representative of the full range of degree of variability, as determined by CVs (Table S2), from highly variable to most stable (Table 5).

**Table 3:** Pearson's correlation coefficients of urinary metabolite intensities between hours of collection on Day 1.

	Hour 1	Hour 3	Hour 7	Hour 9	Hour 11	Hour 14
Hour 1	1.00	0.81	0.74	0.74	0.73	0.73
Hour 3	0.81	1.00	0.82	0.80	0.81	0.76
Hour 7	0.74	0.82	1.00	0.84	0.81	0.82
Hour 9	0.74	0.80	0.84	1.00	0.81	0.82
Hour 11	0.73	0.81	0.81	0.81	1.00	0.83
Hour 14	0.73	0.76	0.82	0.82	0.83	1.00

**Table 4:** Pearson's correlation coefficients of plasma metabolite intensities between hours of collection on Day 1.

	Hour 1	Hour 3	Hour 7	Hour 9	Hour 11	Hour 14
Hour 1	1.00	0.93	0.93	0.93	0.93	0.94
Hour 3	0.93	1.00	0.91	0.92	0.91	0.94
Hour 7	0.93	0.91	1.00	0.94	0.94	0.93
Hour 9	0.93	0.92	0.94	1.00	0.95	0.93
Hour 11	0.93	0.91	0.94	0.95	1.00	0.93
Hour 14	0.94	0.94	0.93	0.93	0.93	1.00

**Table 5:** Coefficients of variation for blood metabolites representing the full range of variability.

Metabolite	Meal^a^	Hour^b^	Day^c^
Glucosylceramide	0.1370	0.1352	0.1246
Glucose-6-Phosphate	0.1065	0.1253	0.1099
Citric acid	0.1017	0.0902	0.0848
Cholesterol	0.0766	0.0766	0.0723
Palmitic acid	0.0513	0.0497	0.0496

a Meal CVs were calculated using pre and post breakfast samples from each day.

b Hour CVs were calculated using all values from Day 1.

c Day CVs were calculated pre-breakfast values from each day.

### Factors most influential to changes in metabolite intensities

To further understand the features that modify metabolite intensities among subjects, we conducted differential analyses to identify which factor(s) significantly influence metabolite intensities. The VCA characterized the relative contribution of each temporal factor but did not address the actual magnitude of the effect of each factor and its significance. The effects of meals and time-of-day were significant for a large number of urinary metabolites but day-to-day variation was minimal with only few metabolites differing significantly across days especially in blood (Table 6). Thus, we found that meals were a significant source of variability in some urine metabolites, but that meals had minimal to no influence on blood metabolites. In general, day effects (as assessed on three days) were not statistically significant, which confirmed the minimal day-to-day variability we observed in the VCA results. However, time-of-day appeared to be a significant factor in urine while its effect on blood metabolome was minimal. The time-of-day effects were partially attributable to meal effects. Taken together, meals and time-of-day appear to be important factors to consider for urinary metabolites and to a lesser degree for blood metabolites.

**Table 6:** Number and percentage (%) of significantly changed metabolites (FDR<0.05) in blood and urine by three factors (meal, day, hour).

	Urine	Blood
Factor	Number (%)	Number (%)
Meal		
Day 1 (+1 &+3)	67 (23)	0
Day 1 (+9 &+11)	20 (7)	0
Day 2 (+1 &+3)	34 (11)	7 (6)
Day 3 (+1 &+3)	39 (13)	1 (0.8)
Hour	135 (46)	11 (9)
Day	2 (0.7)	1 (0.8)

There were a total of 294 metabolites identified in urine and 121 in blood.

## Discussion

While extant studies support the contention that metabolomics has enormous potential to provide insight into physiological as well as pathophysiological processes in human biology with tremendous clinical applicability, the limitations of this technique especially with respect to clinical translation, as with other omics technologies [5], are conceivably manifold. Most such limitations are related to the fact that metabolic processes, more than genes and proteins, are by nature influenced by exogenous as well as endogenous stimuli, for example: meals, medications, menstrual cycles, and circadian rhythms [8], [19]–[23]. While several studies in humans and animals evaluated the influence of these factors on the metabolome, we could find no published studies performed on human subjects in a tightly controlled dietary environment, as would occur in an inpatient clinical research center, such that highly accurate conclusions can be drawn. The current study of plasma and urine metabolome variability was undertaken in a setting of extreme consistency of human subjects in such an inpatient facility in the time scale of hours and days. While this extreme of consistency would of course not occur in the everyday clinical setting, it is necessary to perform this type of study to draw conclusions about which factors of the external environment, particularly parameters related to sample collection, contribute to human metabolome variability and how the metabolome is altered as a result of these factors.

The observed variability in our data can be explained by, in general terms, (1) biological and (2) technical variation. Biological variation reflects the intrinsic properties of a biological system, such as genetic background, gender, age, and state of health. In contrast, technical variability represents non-biological variability such as sampling or technical issues, which often cannot be explicitly pinpointed. Sources of such technical variation include, but are not limited to, measurement errors associated with sample collection (unlikely due to the extreme consistency in this process), sample preparation for chemical analysis, sample injection time and order in the mass spectrometer, chromatographic separation, LC-MS data acquisition and instrument drift, and data processing. While we found minimal systemic inconsistency among batches run on the analytical equipment, there was a large component of residual variability, which resulted from other technical sources other than temporal and mealtime parameters, of the metabolome in all samples. While residual variability will never be completely eliminated, the relatively large proportion of the variability attributed to residual variation (in relation to biological variation), even in such a tightly controlled environment and state-of-the-art analytical laboratories as were used in this study, emphasizes the importance of using standardized and consistent methods of collecting, processing and analyzing samples to minimize as much as possible this source of variation. On the other hand, mealtime variability was relatively low, particularly in blood as compared to urine, indicating high utility of metabolomics for biomarker discovery.

It is important to note that this study was conducted to specifically assess variability in the metabolome as a function of temporal and mealtime parameters and not to identify metabolomic changes associated with any particular disease state or specific biologic parameter. Thus, we utilized a mixed subject pool of healthy subjects with ADPKD as well as healthy volunteers to broaden our results to the general population; however the subject-to-subject variation due to biological differences among subjects (which included but was not limited to ADPKD state) was partitioned separately as a “subject” random effect component in the VCA. It should also be pointed out that the study was not powered to specifically dissect variability attributable to each of intrinsic biological properties, such as genetic mutation of ADPKD, gender, age, race, while controlling for each other's confounding effects. Despite of the lack of statistical power, we evaluated variability separately and show that the individuals with ADPKD showed qualitatively similar results to the disease-free healthy subjects as well as the combined cohort with respect to the temporal variability, suggesting excellent applicability of metabolomics to disease biomarker studies.

Interestingly, urine showed more biological variability than blood, emphasizing the need for more urine samples as compared to blood samples in a well designed metabolomics biomarker study, consistent with a previous animal study from our laboratory [24]. In addition, both blood and urine variability was higher after the morning meal than the evening meal, perhaps reflecting a more pronounced influence on homeostasis when food is consumed after an 8 hr fast as compared to a shorter inter-meal time interval during daytime which may be due to circadian influences independent of meals changes the metabolome. It was also evident that there were a considerably higher number of significantly changed metabolites in urine as compared to blood, with the highest number being a function of time-of-day and mealtime. It was also apparent that there was less variation after the evening meal than the morning, indicating that post-breakfast sampling should be avoided. These data stress the need to be consistent in collection times, and that it may be best to consider fasting samples (which includes mealtimes) when evaluating the urinary metabolome in particular.

Other studies have evaluated the effects of dietary interventions on the human metabolome. In one study, a cohort of 10 healthy volunteers was admitted to a clinical research center for 2 wk of dietary standardization, and urine and plasma metabolomes were measured daily by NMR spectroscopy [7]. These data showed that a standardized diet that lasts a single day is likely sufficient to provide all of the normalization that can be achieved in the human metabolome, and is therefore consistent with our findings of high day-to-day reproducibility. However, a separate study of urine metabolomics in healthy subjects [25] showed a trend towards reduced inter- and intra-individual variation during 3 days of diet standardization. In another study [26], the effect of multiple interventions on metabolomic parameters were evaluated; this study demonstrated that such challenges (extended fasting, glucose and lipid tolerance tests, controlled meals, physical exercise, and physiological stress) increase metabolite variability between volunteers, allowing discrete metabotypes to be identified that would not be seen in normal fasting conditions. As far as examination of the effects of a single dietary intervention on the metabolome, another group in several studies evaluated the effect of the urine metabolome after a single intake of cocoa and found significant changes at 24 hours, inferring that the strategies which they used can dissect the complex relations between the consumption of phytochemicals and their expected effects on human health [27], [28]. Although it was not the purpose of this study, we have shown that for example that glucose and other sugars as well as phospholipids are changed in the post-prandial state (Table S2). Furthermore, citric acid was found to be relatively sensitive to meal difference compared to other metabolites (Table 5). Thus it is important to standardize meals when studying TCA cycle involved diseases.

While the urinary metabolome exhibited considerablly more biological variability than blood in the current study, others have also evaluated the urine metabolome with respect to gender, age, and diet. One group found that metabolites related to mitochondrial energy metabolism helped differentiate gender and age, while dietary components and circadian rhythm metabolites were related to time of day [19]. Another group also used NMR-based techniques to generate models differentiating subjects according to gender and age [29], and another showed that the urine metabolome, in contrast to that in the plasma and saliva, can reflect acute dietary intake [20]. However, none of these studies evaluated relative sources of varbiability; this is an unique aspect of our work and is very germane to clinical translation of any metabolomics study.

There are several studies, performed over a much longer time frame than the current study, that confirm the existence of a stable part of the metabolome that appears in the urine and which seems to be specific to the individual subject. Bernini et al, in a study for which the time scale was years rather than hours and days as we have evaluated, showed that an invariate part of the metabolome was stable over this long period [9]. Assfalg et al also demonstrated an invariate portion of the metabolome characteristic of each person in urine samples taken over a period of 3 months [10]. However, while consistent with our findings, neither of these studies reported hourly or daily changes, or at specific changes as a function of mealtimes in a controlled environment. Heinzmann et al [11] performed an exhaustive study evaluating the human urinary metabolome in 7 individuals in a 7-day controlled diet, although unlike our study these individuals were not admitted to a clinical study center. Consistent with our study, these investigators found marked differences in the individual metabolomes such that each individual possessed his/her own “metabolic phenotype”; the authors made the suggestion, based on their data, that individuals should serve as their own controls. In another study comparing urine, plasma, and saliva on a standard diet [20], it was found that urine was more sensitive in detecting dietary differences than the other biofluids, consistent with our findings in the present study.

In summary, our study utilizing volunteer human subjects in a tightly controlled environment provides for the first time a framework in which to view the data obtained from a well designed and consistently executed metabolomics experiment. While the day-to-day variability was minimal, there are meal and collection time effects to be considered, and these are more pronounced in urine than blood. Given the ready availability of LC-MS techniques in many universities, clinical and commercial laboratories, and the accepted power of metabolomics for clinical translational research, there will likely be many more studies using this technique in the near future. While our study confirms the utility of metabolomics for both blood and urine analysis for discovery of disease targets and biomarkers, the data shown here inject a cautionary note into the design of future metabolomics studies, as has been highlighted in a recent study [5]. For example, based on our data presented here, a well-designed study biofluid metabolomics should have the following characteristics: (1) the study should be designed to avoid systematic errors by minimizing bias by controlling for extraneous variables such as meals or time of day of sample collection; (2) the study should have broad applicability (i.e., the results hold for more than the subjects tested in a study); (3) the study should have sufficient statistical power to test the effect of the parameter of interest (i.e. disease); (4) the study should attempt to minimize technical variability by optimizing analytical tools; and (5) it would be prudent to utilize fasting blood samples, and, if such samples were not feasible, random pre-meal samples obtained during midday would be preferable over post-meal samples to minimize bias by extraneous variables. Keeping these concepts in mind, in both design and interpretation of metabolomics data, will enhance the already considerable utility of this growing field.

## Supporting Information

Figure S1 **Distribution of log_2_ transformed intensities of compounds in each urine reference sample across six LC-MS plates.** Data from urine of 3 ADPKD and 3 control patients. 294 compounds were detected in all urine samples on all plates and used in the analysis.
(TIF)

Figure S2 **Distribution of log_2_ transformed intensities of compounds in each plasma reference sample across six LC-MS plates.** Data from plasma of 3 ADPKD and 3 control patients. 121 compounds were detected in all plasma samples on all plates and used in the analysis.
(TIF)

Figure S3 **Distribution of coefficients of variations (CVs) of metabolites calculated using log_2_ transformed intensity values in urine across six plates for six reference samples.**
(TIF)

Figure S4 **Distribution of coefficients of variations (CVs) of metabolites calculated using log_2_ transformed intensity values in plasma across six plates for six reference samples.**
(TIF)

Figure S5 **Distribution of CVs of 294 urinary metabolites (left) and 121 blood metabolites (right) across three days in fasting and post-breakfast samples.**
(TIF)

Figure S6 **Time courses of intensities for three highly variable metabolites in urine of healthy subjects throughout Day 1.** These three metabolites had the highest CVs calculated using all observations on Day 1. Each line represents one person.
(TIF)

Table S1 **Characteristics of the Subject Population.**
(DOC)

Table S2 **Temporal and mealtime variability of selected blood metabolites.**
(XLS)

File S1 **Comparison between ADPKD and normal subjects.**
(DOCX)

File S2 **Raw and normalized LC/TOF-MS data, subject characteristics, and run order for (a) plasma and (b) urine.**
(XLSX)
