# Supplementary figures and images for: Mealtime, Temporal, and Daily Variability of the Human Urinary and Plasma Metabolomes in a Tightly Controlled Environment
Source: PLoS One. 2014 Jan 24;9(1):e86223. doi: 10.1371/journal.pone.0086223 (PMC3901684; doi:10.1371/journal.pone.0086223)

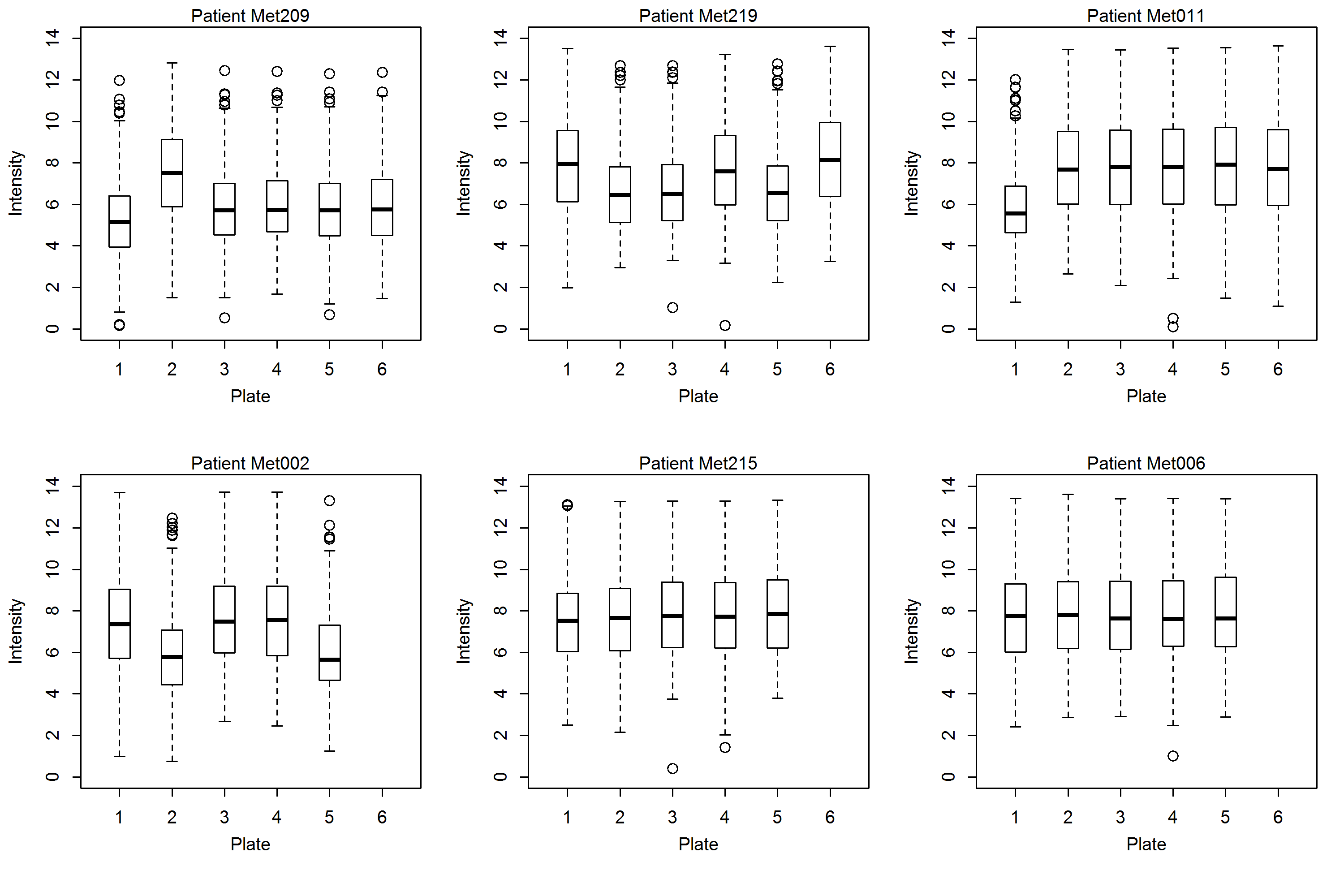

Supplement: Figure S1 — Distribution of log2 transformed intensities of compounds in each urine reference sample across six LC-MS plates. Data from urine of 3 ADPKD and 3 control patients. 294 compounds were detected in all urine samples on all plates and used in the analysis. (TIF) [file pone.0086223.s001.tif]

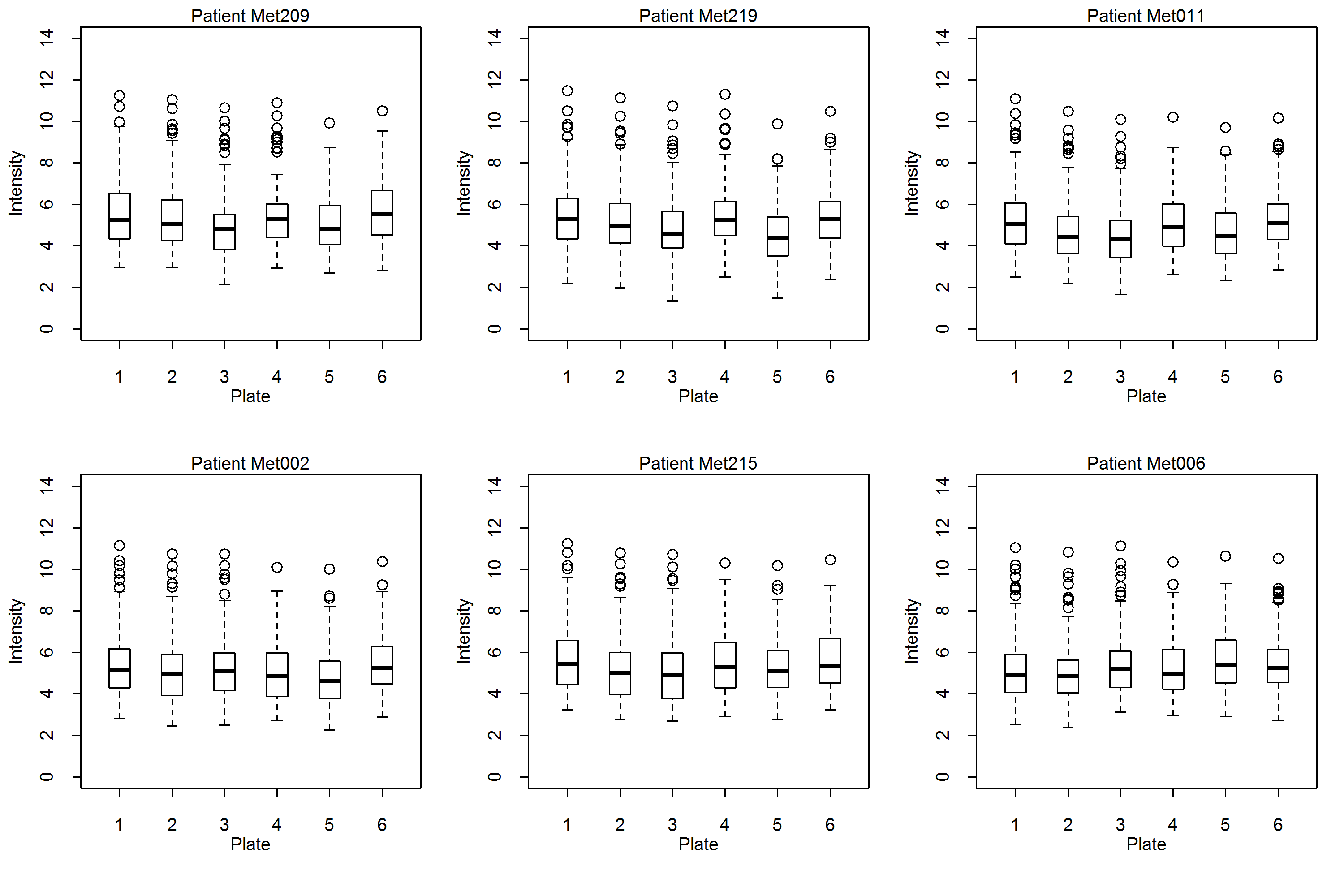

Supplement: Figure S2 — Distribution of log2 transformed intensities of compounds in each plasma reference sample across six LC-MS plates. Data from plasma of 3 ADPKD and 3 control patients. 121 compounds were detected in all plasma samples on all plates and used in the analysis. (TIF) [file pone.0086223.s002.tif]

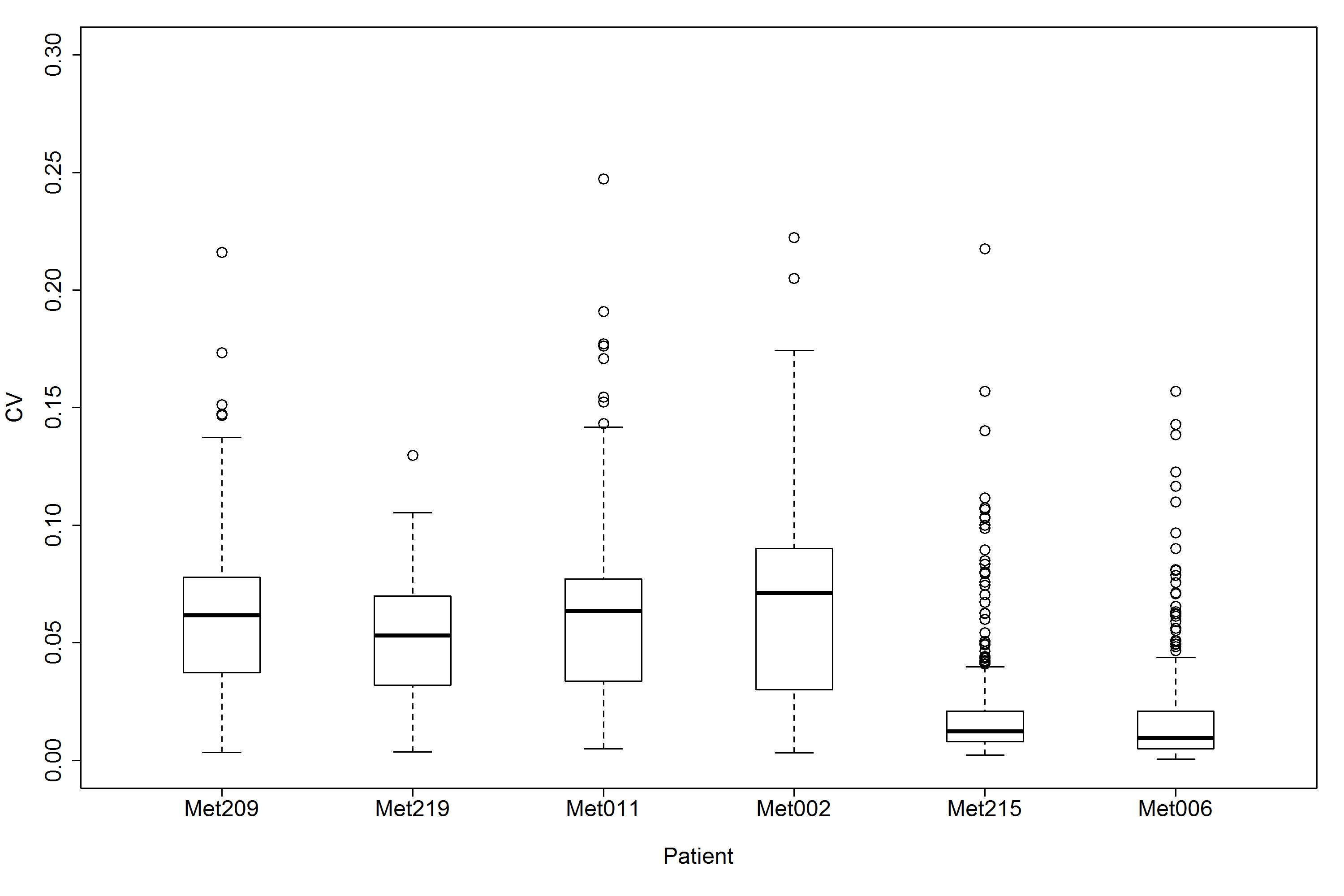

Supplement: Figure S3 — Distribution of coefficients of variations (CVs) of metabolites calculated using log2 transformed intensity values in urine across six plates for six reference samples. (TIF) [file pone.0086223.s003.tif]

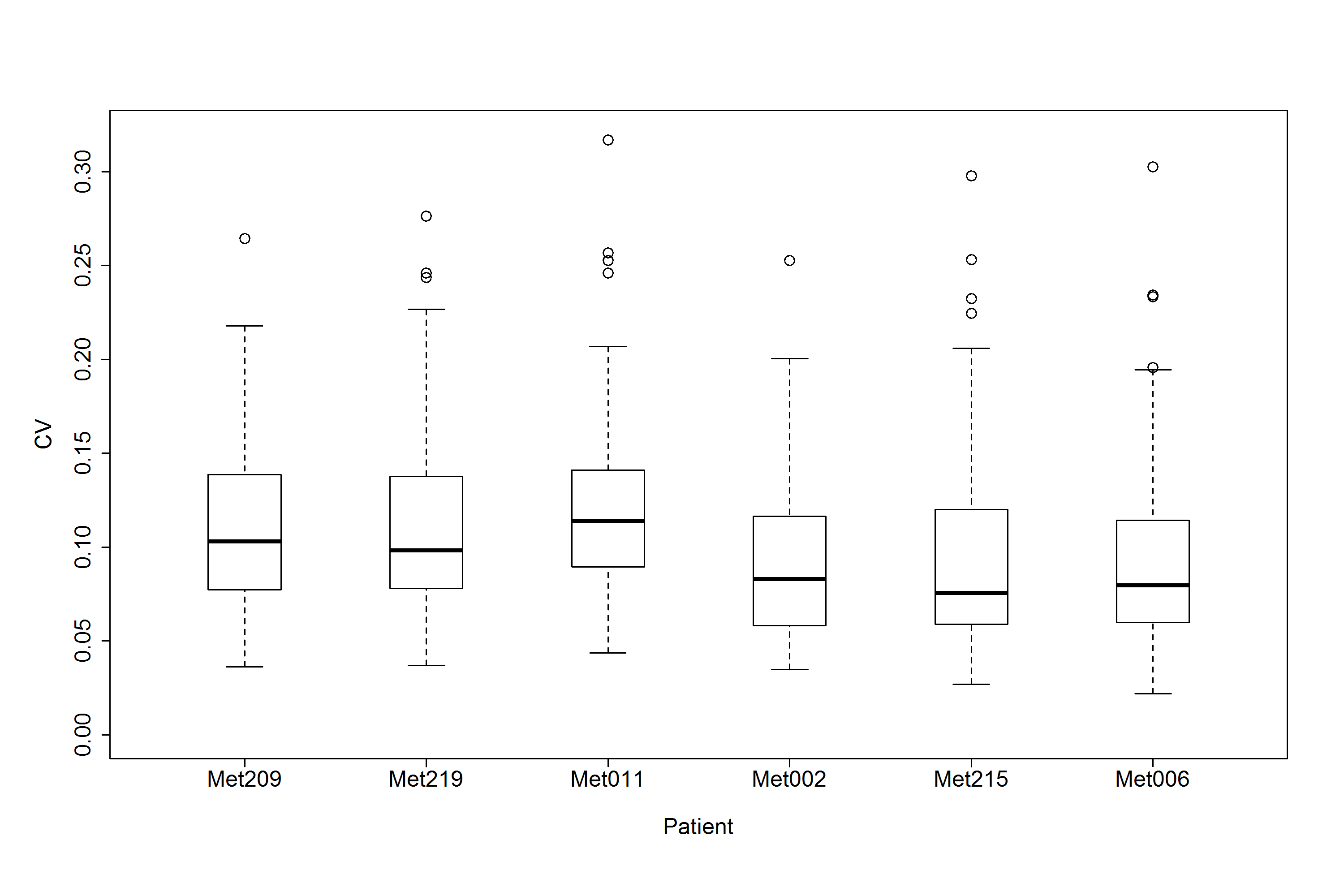

Supplement: Figure S4 — Distribution of coefficients of variations (CVs) of metabolites calculated using log2 transformed intensity values in plasma across six plates for six reference samples. (TIF) [file pone.0086223.s004.tif]

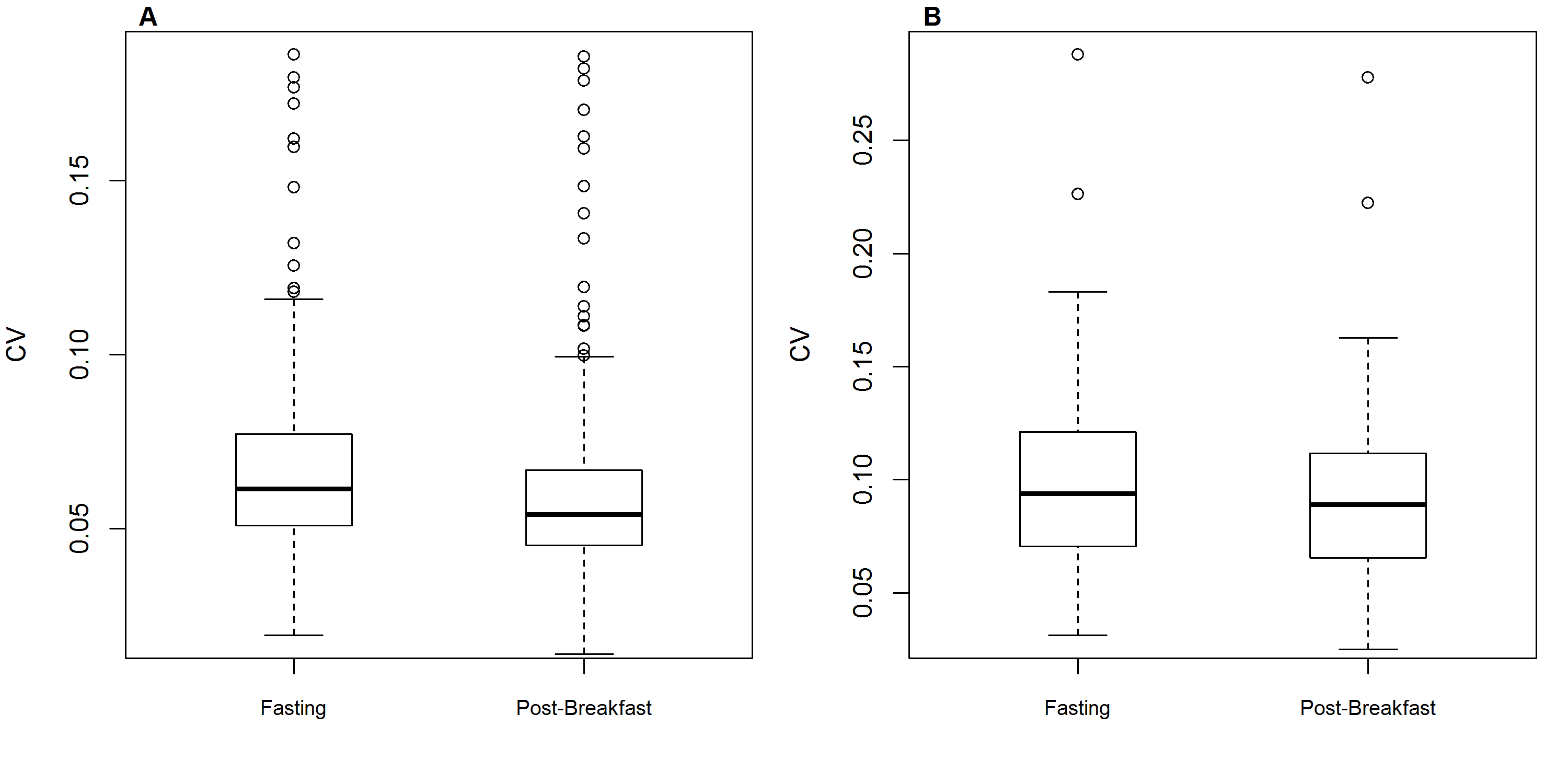

Supplement: Figure S5 — Distribution of CVs of 294 urinary metabolites (left) and 121 blood metabolites (right) across three days in fasting and post-breakfast samples. (TIF) [file pone.0086223.s005.tif]

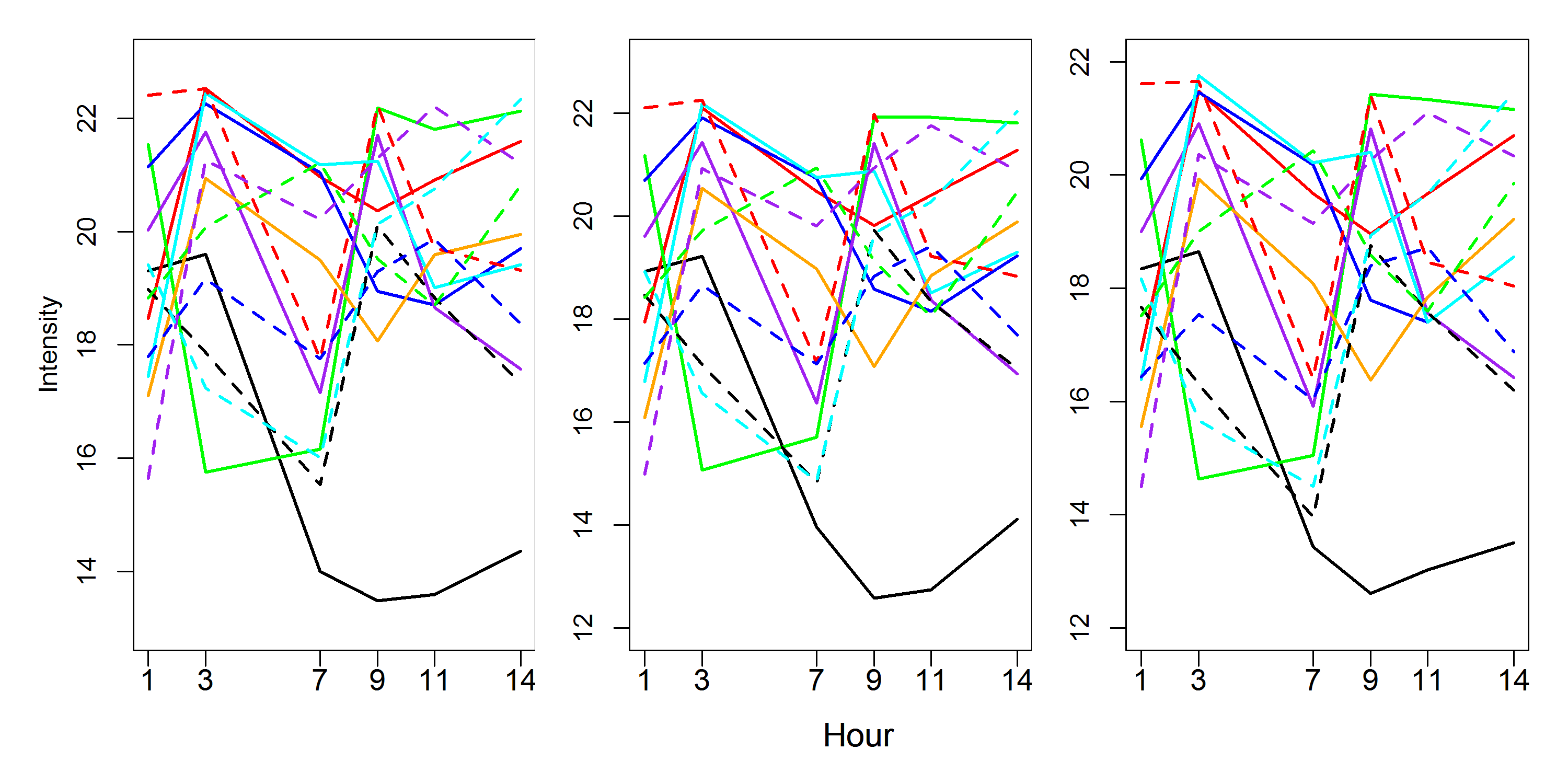

Supplement: Figure S6 — Time courses of intensities for three highly variable metabolites in urine of healthy subjects throughout Day 1. These three metabolites had the highest CVs calculated using all observations on Day 1. Each line represents one person. (TIF) [file pone.0086223.s006.tif]
