# Supplementary material for: Mealtime, Temporal, and Daily Variability of the Human Urinary and Plasma Metabolomes in a Tightly Controlled Environment
Source: PLoS One. 2014 Jan 24;9(1):e86223. doi: 10.1371/journal.pone.0086223 (PMC3901684; doi:10.1371/journal.pone.0086223)
Supplement: Table S1 — Characteristics of the Subject Population. (DOC) [file pone.0086223.s007.doc]

Table S1. Characteristics of the Subject Population

| Gender | Race | Age | PKD status | ht (cm) | wt (kg) | BMI  (kg/m^2^**)** | Serum  Creatinine  (mg/dL) | MDRD GFR  (mL/min/1.73 m^2^) |
| --- | --- | --- | --- | --- | --- | --- | --- | --- |
|  |  |  |  |  |  |  |  |  |
| | M | | --- | | M | | F | | F | | M | | M | | M | | M | | M | | M | | M | | M | | F | | F | | F | | F | | F | | F | | F | | F | | M | | M | | M | | M | | F | | F | | | W | | --- | | W | | W | | W | | AA | | AA | | W | | W | | AA | | AA | | W | | W | | W | | W | | W | | W | | W | | W | | AA | | AA | | Asian | | W | | AA | | AA | | AA | | AA | | | 28 | | --- | | 28 | | 28 | | 27 | | 22 | | 22 | | 27 | | 27 | | 24 | | 25 | | 41 | | 41 | | 50 | | 54 | | 24 | | 25 | | 52 | | 52 | | 49 | | 44 | | 34 | | 37 | | 27 | | 23 | | 23 | | 26 | | | PKD | | --- | | Healthy | | PKD | | Healthy | | PKD | | Healthy | | PKD | | Healthy | | PKD | | Healthy | | PKD | | Healthy | | PKD | | Healthy | | PKD | | Healthy | | PKD | | Healthy | | PKD | | Healthy | | PKD | | Healthy | | PKD | | Healthy | | PKD | | Healthy | | | 182.9 | | --- | | 172.7 | | 165.0 | | 161.0 | | 187.1 | | 188.0 | | 188.5 | | 174.2 | | 175.5 | | 173.0 | | 182.5 | | 169.0 | | 173.0 | | 167.6 | | 174.6 | | 174.4 | | 165.0 | | 153.0 | | 174.0 | | 153.0 | | 167.5 | | 188.0 | | 179.1 | | 187.5 | | 178.1 | | 155.0 | | | 92.0 | | --- | | 89.4 | | 60.2 | | 51 | | 90.1 | | 98.0 | | 99.2 | | 89.4 | | 61.6 | | 62.4 | | 92.1 | | 70.1 | | 70.3 | | 61.1 | | 59.9 | | 59.1 | | 56.2 | | 50.9 | | 71.8 | | 61.6 | | 67.5 | | 83.1 | | 86.9 | | 94.8 | | 67.6 | | 66.8 | | | 27.5 | | --- | | 30.0 | | 22.1 | | 19.7 | | 25.7 | | 27.7 | | 27.9 | | 29.5 | | 20.0 | | 20.8 | | 27.7 | | 24.5 | | 23.5 | | 21.8 | | 19.7 | | 19.5 | | 20.6 | | 21.7 | | 23.7 | | 26.3 | | 24.1 | | 23.5 | | 27.1 | | 27.0 | | 21.3 | | 27.8 | | | 1.09 | | --- | | 0.90 | | 0.58 | | 0.67 | | 0.93 | | 1.24 | | 0.88 | | 1.03 | | 0.94 | | 0.83 | | 0.87 | | 0.80 | | 1.03 | | 0.77 | | 0.70 | | 0.72 | | 0.82 | | 0.60 | | 0.80 | | 0.75 | | 0.86 | | 0.87 | | 1.25 | | 1.20 | | 0.82 | | 0.82 | | | 81 | | --- | | 100 | | 124 | | 106 | | 123 | | 88 | | 104 | | 87 | | 120 | | 137 | | 97 | | 107 | | 57 | | 78 | | 103 | | 99 | | 73 | | 105 | | 92 | | 102 | | 102 | | 99 | | 84 | | 91 | | 105 | | 102 | |  | |

54% M 58% W

Mean ± SD: 33 ± 10.9 173.4 ± 10.6 73.6 ± 15.5 24.3 ± 3.3 0.88 ± 0.18 99 ± 17

PKD, polycystic kidney disease; ht, height; wt, weight; BMI, body mass index; MDRD, modification of diet in renal disease; GFR, glomerular filtration rate; M, male; F, female; W, white; AA, African American.
