# Supplementary material for: Mealtime, Temporal, and Daily Variability of the Human Urinary and Plasma Metabolomes in a Tightly Controlled Environment
Source: PLoS One. 2014 Jan 24;9(1):e86223. doi: 10.1371/journal.pone.0086223 (PMC3901684; doi:10.1371/journal.pone.0086223)
Supplement: File S1 — Comparison between ADPKD and normal subjects. (DOCX) [file pone.0086223.s009.docx]

File S1, Fig. 1(a). Distributions of the proportion of variance of 121 blood metabolites explained by meals effects, between patient variability and residual variability for healthy (i.e., normal) subjects (left column) and PKD subjects (right column). Meal effects were represented by pre and post breakfast samples on Day 1 (top row) and Day 2 (bottom row).

File S1, Fig. 1(b). Distributions of the proportion of variance of 121 blood metabolites explained by meals effects, between patient variability and residual variability for healthy (i.e., normal) subjects (left column) and PKD subjects (right column). Meal effects were represented by pre and post breakfast samples on Day 3 (top row) and pre and post dinner samples on Day 1 (bottom row).

File S1, Fig 2. Distributions of the proportion of variance of 121 blood metabolites explained by time of day (hour) effects (top row) and day-to-day effects (bottom row), and between patient variability and residual variability for healthy (i.e., normal) subjects (left column) and PKD subjects (right column).

File S1, Fig. 3(a). Distributions of the proportion of variance of 294 urinary metabolites explained by meals effects, between patient variability and residual variability for healthy (i.e., normal) subjects (left column) and PKD subjects (right column). Meal effects were represented by pre and post breakfast samples on Day 1 (top row) and Day 2 (bottom row).

File S1, Fig. 3(b). Distributions of the proportion of variance of 294 urinary metabolites explained by meals effects, between patient variability and residual variability for healthy (i.e., normal) subjects (left column) and PKD subjects (right column). Meal effects were represented by pre and post breakfast samples on Day 3 (top row) and pre and post dinner samples on Day 1 (bottom row).

File S1, Fig. 4. Distributions of the proportion of variance of 294 urinary metabolites explained by time of day (hour) effects (bottom row) and day-to-day effects (top row), and between patient variability and residual variability for healthy (i.e., normal) subjects (left column) and PKD subjects (right column).

| **NORMAL SUBJECTS ONLY**  File S1, Table 1. Proportion (mean ± SD) of variance attributable to each source of variation in metabolite intensity across all metabolites. | | | | | | | |
| --- | --- | --- | --- | --- | --- | --- | --- |
|  |  | Within-Day Variability | | | | | Between-Day Variability |
|  | Source of variance | Meal effects | | | | Time of day effects | Day-to-Day effects |
|  |  | Day 1  (+1 &+3) | Day 1  (+9 &+11) | Day 2  (+1 &+3) | Day 3  (+1 &+3) | Day 1  (hours +1 to +14) | Days 1-3  (only +1 hour) |
| Urine | Meal | 0.100±0.18 | 0.058±0.11 | 0.099±0.16 | 0.076±0.16 | 0.109±0.16 | 0.030±0.05 |
|  | Patient | 0.301±0.27 | 0.298±0.28 | 0.294±0.27 | 0.210±0.25 | 0.226±0.22 | 0.190±0.19 |
|  | Residuals | 0.599±0.30 | 0.643±0.30 | 0.607±0.254 | 0.713±0.27 | 0.664±0.27 | 0.780±0.20 |
| Blood | Meal | 0.010±0.02 | 0.013±0.04 | 0.044±0.08 | 0.018±0.04 | 0.026±0.05 | 0.016±0.03 |
|  | Patient | 0.400±0.28 | 0.349±0.31 | 0.417±0.22 | 0.303±0.32 | 0.330±0.271 | 0.319±0.25 |
|  | Residuals | 0.593±0.28 | 0.638±0.31 | 0.539±0.22 | 0.679±0.318 | 0.644±0.278 | 0.665±0.25 |

| **PKD SUBJECTS ONLY**  File S1, Table 2. Proportion (mean ± SD) of variance attributable to each source of variation in metabolite intensity across all metabolites. | | | | | | | |
| --- | --- | --- | --- | --- | --- | --- | --- |
|  |  | Within-Day Variability | | | | | Between-Day Variability |
|  | Source of variance | Meal effects | | | | Time of day effects | Day-to-Day effects |
|  |  | Day 1  (+1 &+3) | Day 1  (+9 &+11) | Day 2  (+1 &+3) | Day 3  (+1 &+3) | Day 1  (hours +1 to +14) | Days 1-3  (only +1 hour) |
| Urine | Meal | 0.060±0.12 | 0.030±0.08 | 0.079±0.17 | 0.063±0.12 | 0.077±0.11 | 0.040±0.07 |
|  | Patient | 0.498±0.23 | 0.323±0.21 | 0.196±0.23 | 0.349±0.26 | 0.265±0.17 | 0.431±0.19 |
|  | Residuals | 0.442±0.22 | 0.646±0.21 | 0.724±0.27 | 0.589±0.28 | 0.658±0.20 | 0.529±0.19 |
| Blood | Meal | 0.039±0.06 | 0.016±0.05 | 0.049±0.08 | 0.007±0.04 | 0.032±0.04 | 0.012±0.04 |
|  | Patient | 0.324±0.29 | 0.428±0.27 | 0.327±0.28 | 0.449±0.26 | 0.304±0.26 | 0.256±0.24 |
|  | Residuals | 0.637±0.29 | 0.553±0.27 | 0.623±0.27 | 0.544±0.26 | 0.663±0.25 | 0.731±0.25 |

| File S1, Table 3. Number and percentage (%) of significantly changed metabolites (FDR < 0.05) in blood and urine by three factors (meal, day, hour). There were a total of 294 metabolites identified in urine and 121 in blood. | | |
| --- | --- | --- |
|  | **ALL SUBJECTS** | |
|  | Urine | Blood |
| Factor | Number (%) | Number (%) |
| Meal |  |  |
| Day 1 (+1 &+3) | 67 (23) | 0 |
| Day 1 (+9 &+11) | 20 (7) | 0 |
| Day 2 (+1 &+3) | 34 (11) | 7 (6) |
| Day 3 (+1 &+3) | 39 (13) | 1 (0.8) |
| Hour | 135 (46) | 11 (9) |
| Day | 2 (0.7) | 1 (0.8) |
|  | **Normal SUBJECTS** | |
|  | Urine | Blood |
| Factor | Number | Number |
| Meal |  |  |
| Day 1 (+1 &+3) | 23 | 0 |
| Day 1 (+9 &+11) | 44 | 0 |
| Day 2 (+1 &+3) | 14 | 0 |
| Day 3 (+1 &+3) | 21 | 0 |
| Hour | 108 | 9 |
| Day | 0 | 0 |
|  | **PKD SUBJECTS** | |
|  | Urine | Blood |
| Factor | Number | Number |
| Meal |  |  |
| Day 1 (+1 &+3) | 0 | 0 |
| Day 1 (+9 &+11) | 30 | 0 |
| Day 2 (+1 &+3) | 21 | 1 |
| Day 3 (+1 &+3) | 26 | 0 |
| Hour | 83 | 6 |
| Day | 5 | 0 |
